# Supplementary material for: Pronounced centennial-scale Atlantic Ocean climate variability correlated with Western Hemisphere hydroclimate
Source: Nat Commun. 2018 Jan 26;9:392. doi: 10.1038/s41467-018-02846-4 (PMC5786086; doi:10.1038/s41467-018-02846-4)
Supplement: Supplementary file 1 — Supplementary Information [file 41467_2018_2846_MOESM1_ESM.pdf]

Supplementary Figures

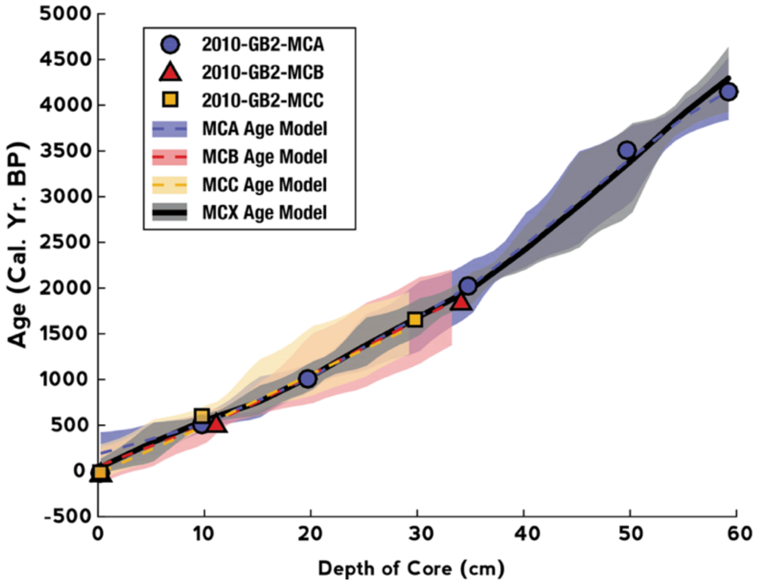

Supplementary Figure 1. Bayesian posteriors of calibrated ages versus depth for MCA (purple), MCB (red), MCC (yellow), and the overall age model (MCX; black) using BACON [1]. The full uncertainty envelopes have been plotted.

Supplementary Figure 2. A demonstration of our uncertainty modeling algorithm where 5000 Mg/Ca time series from MCA are plotted using a bootstrap Monte Carlo framework. We plot the median value and associated 68% and 95% confidence bounds in the second panel. Dates are plotted in grey triangles below.

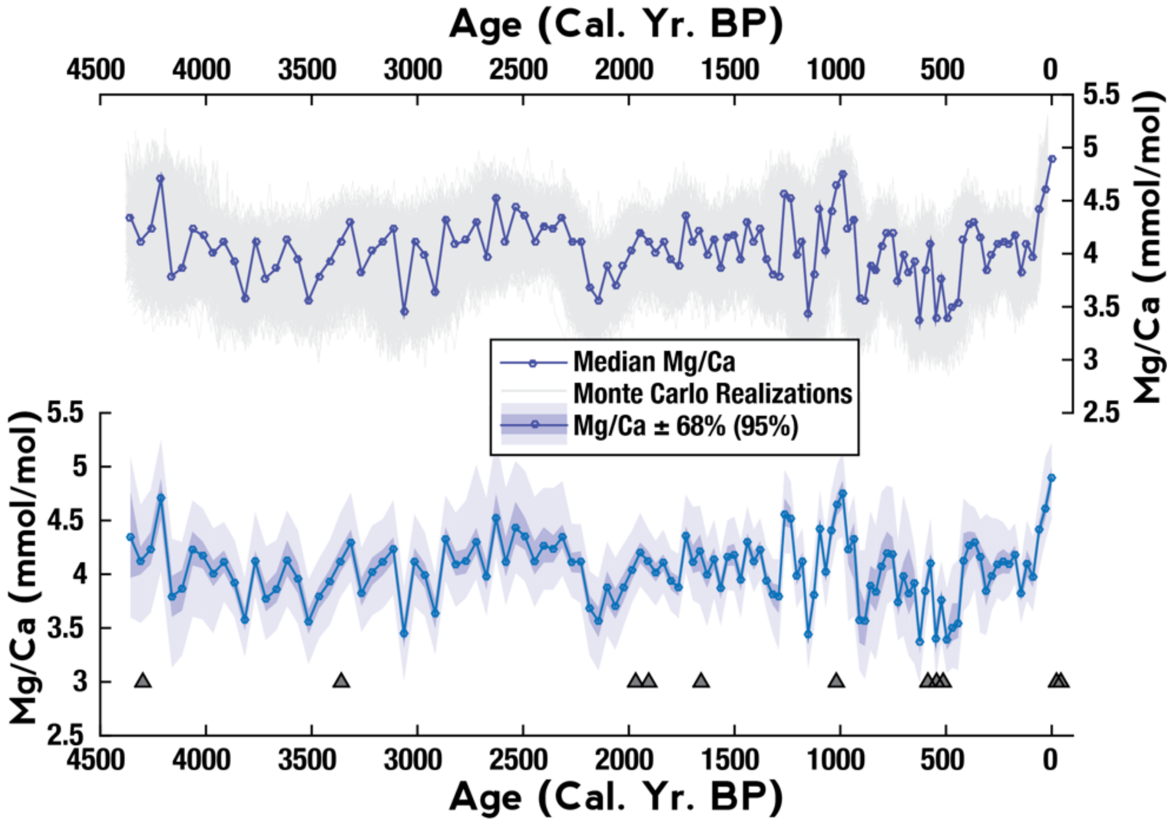

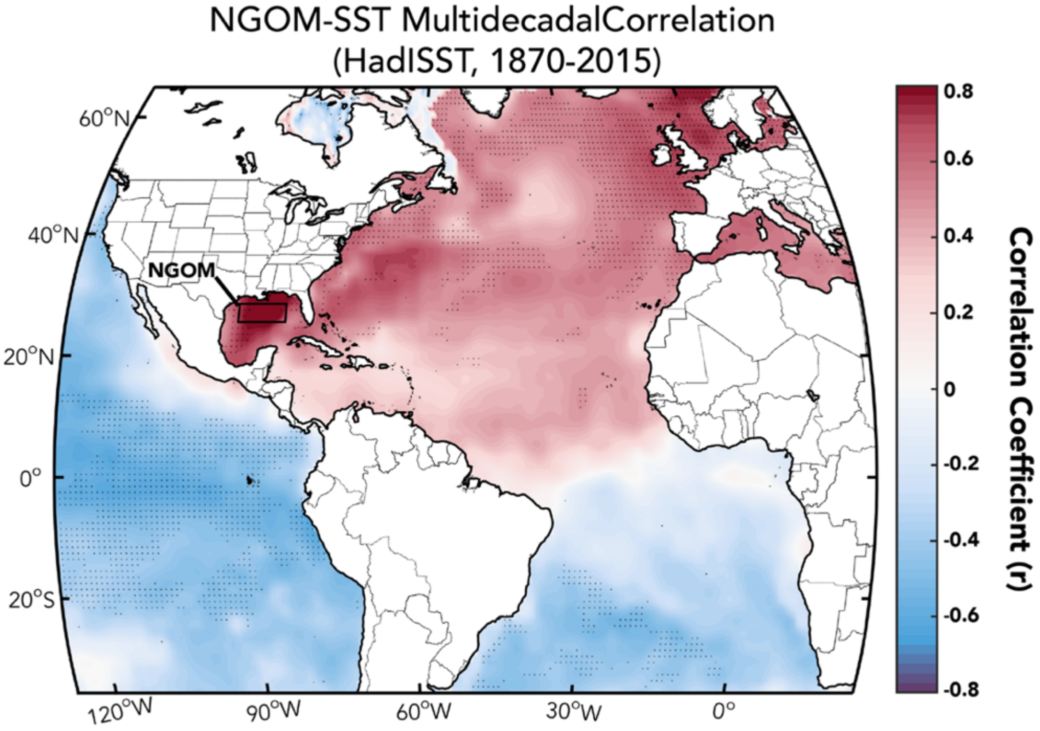

Supplementary Figure 3. Correlation map between northern Gulf of Mexico SSTs (black box) and global oceanic SST using the gridded HadISST monthly dataset. The data were filtered using an 8-year Gaussian filter and detrended prior to correlation to reduce sensitivity to interannual variability. Stippling indicates significance at the 95% level. The map in this figure was generated using the *M_MAP* package in MATLAB.

Supplementary Figure 4. Comparison between correlation analyses performed on MPI-ESM last millennium output using an 8-yr lowpass filter and the 50-150-yr bandpass filter. Correlation analyses were performed as described in the Methods section. Maps in this figure were generated using the *M_MAP* package in MATLAB.

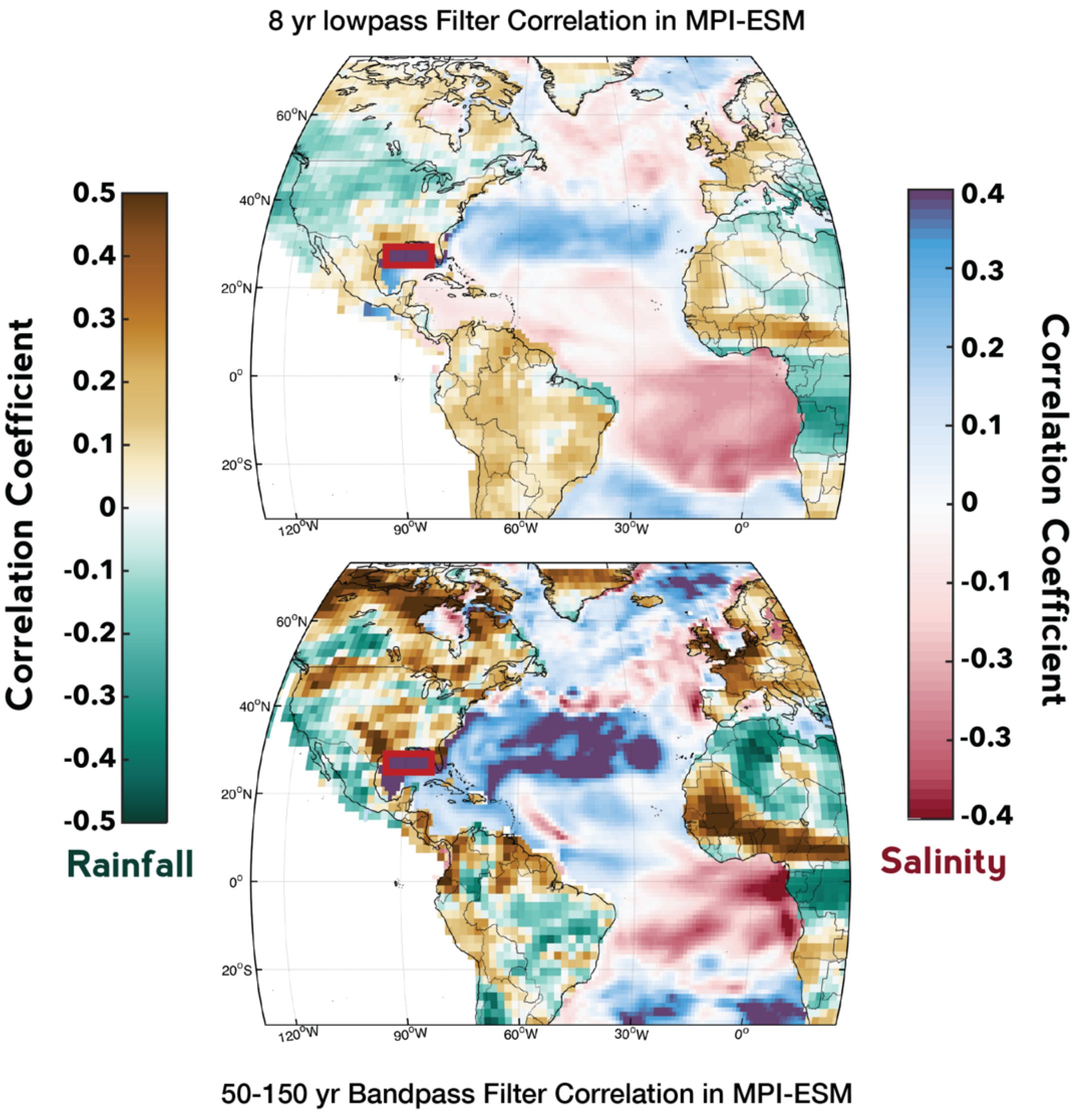

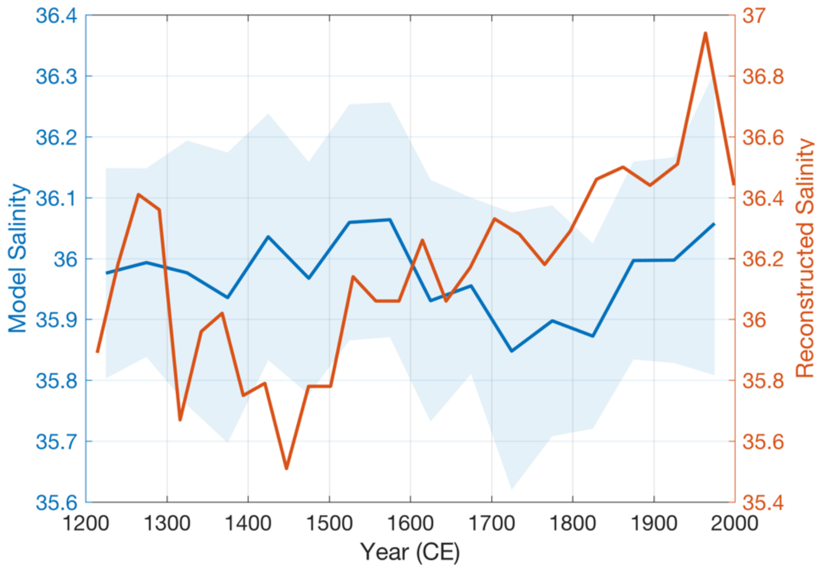

Supplementary Figure 5. For illustrative purposes: comparison between the low-frequency (50-yr running mean with standard error; blue) simulated salinity in the northern Gulf of Mexico over the last millennium from the MPI-ESM and the reconstructed salinity (orange) at the Garrison Basin site.

Supplementary Table

Supplementary Table 1. List of proxies utilized in Figure 1. LIA-mean calculated based on mean state during LIA (1450-1850 AD) relative to the modern era.

Location	Proxy Type	Expectation during LIA based on observations*	Proxy Record during LIA†
**Salinity Proxies**			
Garrison Basin, GOM	*G. ruber* (W) $\delta^{18}O_{sw}$	Fresher	Fresher
Pigmy Basin[2], GOM	*G. ruber* (W) $\delta^{18}O_{sw}$	Fresher	Fresher
Dry Tortugas[3]	*G. ruber* (W) $\delta^{18}O_{sw}$	Fresher	Fresher
Great Bahama Bank[3]	*G. ruber* (W) $\delta^{18}O_{sw}$	Fresher	Fresher
Carolina Slopes[4]	*G. ruber* (W) $\delta^{18}O_{sw}$	Fresher	Fresher
Bermuda[5]	*D. labyrinthiformis* $\delta^{18}O_{sw}$	Fresher	Fresher
Puerto Rico[6]	*M. faveolata* $\delta^{18}O_{sw}$	Fresher	Fresher
Feni Drift[7]	*G. bulloides*	Fresher	Saltier
South Iceland Rise[8]	*G. bulloides*	Fresher/No Change	No Change
Labrador Sea[9]	*T. quinqueloba* $\delta^{18}O_{sw}$	Fresher	Fresher
**Precipitation Proxies**			
Ghana[10]	Authigenic Carbonate $\delta^{18}O$	Dryer	Dryer
Cariaco Basin[11]	Titanium Percent	Dryer	Dryer
Peruvian Andes[12]	Authigenic Carbonate $\delta^{18}O$	Wetter	Wetter
Quelcayya Ice Cap[13]	Ice Core $\delta^{18}O$	Wetter	Wetter
Northern Peru[14]	Stalagmite $\delta^{18}O$	Wetter	Wetter
Northwestern Venezuela[15]	Cyperaceae Pollen, Magnetic Susceptibility	Wetter	Wetter
Central Mexico[16]	Stalagmite $\delta^{18}O$	Wetter	Wetter

Central Mexico[17]	Authigenic Carbonate $\delta^{18}O$	Wetter	Wetter
Yucatan[18]	Stalagmite $\delta^{18}O$	Wetter	Wetter
Southwest US[19]	Tree-rings	Wetter	Wetter
New Mexico[20]	Stalagmite Width	Wetter	Wetter
Southeast US[19]	Tree-rings	Dryer	Dryer

* - Based on Figure 1 correlation analysis

† - Calculated based on the difference in mean of $\delta^{18}O_{sw}$ record from 1450-1850 C.E. and mean from 1850 C.E.-present(/coretop).

Supplementary References

1. Blaauw, M. & Christen, J. A. Flexible Paleoclimate Age-Depth Models Using an Autoregressive Gamma Process. *Bayesian Analysis* **6,** 457–474 (2011).
2. Richey, J. N., Poore, R. Z., Flower, B. P. & Quinn, T. M. 1400 yr multiproxy record of climate variability from the northern Gulf of Mexico. *Geol.* **35,** 423–4 (2007).
3. Lund, D. C. & Curry, W. B. Florida Current surface temperature and salinity variability during the last millennium. *Paleoceanography* **21,** PA2009–15 (2006).
4. Saenger, C., Came, R. E., Oppo, D. W., Keigwin, L. D. & Cohen, A. L. Regional climate variability in the western subtropical North Atlantic during the past two millennia. *Paleoceanography* **26,** PA2206 (2011).
5. Goodkin, N. F., Hughen, K. A., Curry, W. B., Doney, S. C. & Ostermann, D. R. Sea surface temperature and salinity variability at Bermuda during the end of the Little Ice Age. *Paleoceanography* **23,** PA3203 (2008).
6. Kilbourne, K. H. *et al.* Coral windows onto seasonal climate variability in the northern Caribbean since 1479. *Geochem. Geophys. Geosyst.* **11,** n/a–n/a (2010).
7. Richter, T. O., Peeters, F. J. C. & van Weering, T. C. E. Late Holocene (0-2.4kaBP) surface water temperature and salinity variability, Feni Drift, NE Atlantic Ocean. *Quat. Sci. Rev.* **28,** 1941–1955 (2009).
8. Thornalley, D. J. R., Elderfield, H. & McCave, I. N. Holocene oscillations in temperature and salinity of the surface subpolar North Atlantic. *Nature* **457,** 711–714 (2009).
9. Moffa-Sanchez, P., Hall, I. R., Barker, S., Thornalley, D. J. R. & Yashayaev, I. Surface changes in the eastern Labrador Sea around the onset of the Little Ice Age. *Paleoceanography* **29,** 160–175 (2014).
10. Shanahan, T. M. *et al.* Atlantic Forcing of Persistent Drought in West Africa.

Science **324,** 1–4 (2009).
11. Haug, G. H., Hughen, K. A., Sigman, D. M., Peterson, L. C. & Röhl, U. Southward Migration of the Intertropical Convergence Zone Through the Holocene. *Science* **293,** 1304–1308 (2001).
12. Bird, B. W., Abbott, M. B. & Vuille, M. A 2,300-year-long annually resolved record of the South American summer monsoon from the Peruvian Andes. *Proc. Nat. Acad. Sci.* **108,** 8583–8588 (2011).
13. Thompson, L. G., Mosley-Thompson, E., Dansgaard, W. & Grootes, P. M. The Little Ice Age as Recorded in the Stratigraphy of the Tropical Quelccaya Ice Cap. *Science* **234,** 361–364 (1986).
14. Reuter, J. *et al.* A new perspective on the hydroclimate variability in northern South America during the Little Ice Age. *Geophys. Res. Lett.* **36,** L21706–5 (2009).
15. Polissar, P. J. *et al.* Solar modulation of Little Ice Age climate in the tropical Andes. *Proc. Nat. Acad. Sci.* **103,** 8937–8942 (2006).
16. Lachniet, M. S., Bernal, J. P., Asmerom, Y., Polyak, V. J. & Piperno, D. R. A 2400 yr Mesoamerican rainfall reconstruction links climate and cultural change. *Geol.* **40,** 259–262 (2012).
17. Bhattacharya, T. *et al.* Cultural implications of late Holocene climate change in the Cuenca Oriental, Mexico. *Proc. Nat. Acad. Sci.* **112,** 1693–1698 (2015).
18. Medina-Elizalde, M. & Rohling, E. J. Collapse of Classic Maya Civilization Related to Modest Reduction in Precipitation. *Science* **335,** 956–959 (2012).
19. Cook, E. R., Seager, R., Cane, M. A. & Stahle, D. W. North American drought: Reconstructions, causes, and consequences. *Earth Sci. Rev.* **81,** 93–134 (2007).
20. Polyak, V. J. & Asmerom, Y. Late Holocene Climate and Cultural Changes in the Southwestern United States. *Science* **294,** 148–151 (2001).
